# A systemic review of the utility of antituberculosis therapy for presumed tuberculous uveitis

**DOI:** 10.1186/s12879-024-10288-1

**Published:** 2025-01-24

**Authors:** Jemma W. Taylor, Ginevra E. L. Wright, Lyndell L. Lim, Justin T. Denholm

**Affiliations:** 1 Department of Infectious Diseases at the Peter Doherty Institute for Infection and Immunity, University of Melbourne, 792 Elizabeth Street, Melbourne, 3000 Australia; 2 University of Melbourne, Parkville, Australia; 3 Centre for Eye Research Australia, East Melbourne, Victoria, Australia; 4 University of Melbourne, Parkville, VIC Australia; 5 Royal Victorian Eye and Ear Hospital, East Melbourne, Victoria, Australia; 6 Royal Melbourne Hospital, Parkville, VIC Australia; 7 Department of Infectious Diseases at the Peter Doherty Institute for Infection and Immunity, Victorian Tuberculosis Program, Melbourne Health, University of Melbourne, Melbourne, Australia

**Keywords:** Tuberculosis, Ocular tuberculosis, Extrapulmonary tuberculosis, Ophthalmology, Tuberculous uveitis

## Abstract

**Background:**

Uveitis presumed to be secondary to *Mycobacterium tuberculosis* is a rare but potentially blinding condition. Difficulty in making an accurate diagnosis and the low incidence of TB uveitis (TBU) contribute to the lack of evidence regarding the best management of this condition. This systematic review aims to analyse existing research to provide a summary of the literature regarding the utility of TB therapy for the management of TBU.

**Methods:**

This systematic review was prospectively registered on PROSPERO (PROSPERO 2021 CRD42021273379). We searched Medline, Embase and Central databases, and the search was done on 20th June 2023 with an updated literature search.

**Results:**

We included 55 studies and found that the heterogeneity in the methodology of these studies precluded metanalysis, and a narrative analysis was undertaken. Risk of bias analysis was undertaken using the Newcastle–Ottawa scale.

**Conclusions:**

Key findings of this systematic review include multiple systemic biases in the available evidence, and general lack of control for confounding variables. This results in many unanswered questions regarding the utility of TB therapy for TBU and reinforces the need for more data in this area.

**Supplementary Information:**

The online version contains supplementary material available at 10.1186/s12879-024-10288-1.

## Background

Uveitis caused by *Mycobacterium tuberculosis* (TB) infection is a rare condition with significant morbidity [1]. It is difficult to diagnose, and current management guidelines are predominantly consensus opinion based [2]. The incidence of uveitis of any aetiology varies from 30–700/100,000 people depending on geographical region, and accounts for 5–20% of blindness in high income settings and up to 25% of blindness in low-income settings [3]. Establishing a clear aetiology for uveitis can be extremely difficult, with a cause established in only 30–70% of cases [4]. Uveitis can be infective or non-infective/autoimmune, with most diagnoses established by the phenotype of the uveitis as assessed by an expert ophthalmologist with supporting laboratory investigations. Where a laboratory test confirms the diagnoses, this is most often the result of testing of non-ocular samples, most commonly blood.

**Fig. 1:**
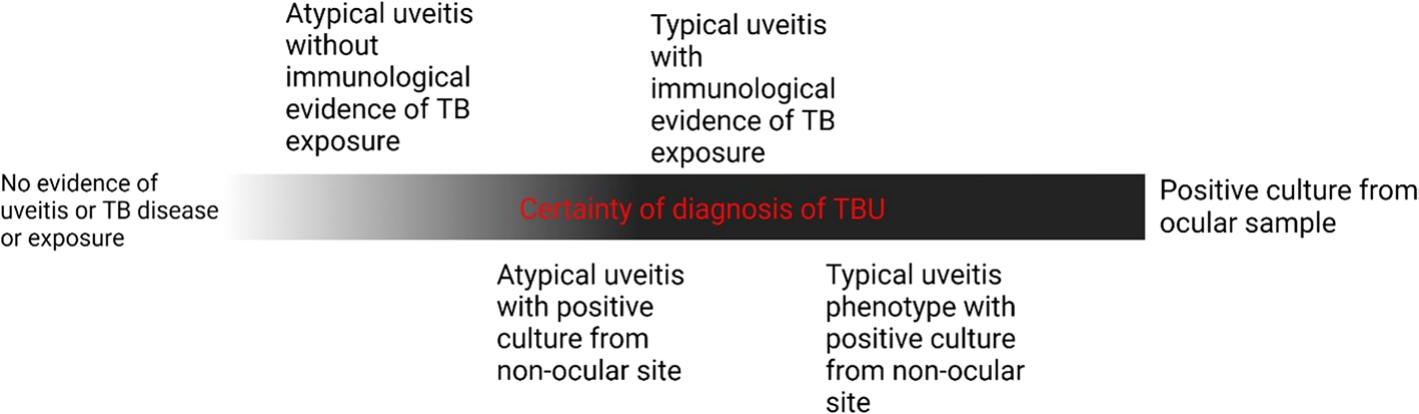
Certainty of diagnosis of TBU

In acute fulminant presentations of uveitis, intraocular sampling may be required to diagnose infections such as Herpes viruses, toxoplasma retinochoroiditis, or endogenous endophthalmitis. Where an aetiology can be established, frequent autoimmune causes include sarcoidosis, HLA-B27 related spondyloarthropathies, and Behçet’s disease [5]. A retrospective case review of 1752 uveitis patients conducted in Australia found that 13.4% of cases of uveitis were infective in aetiology, and of those 20/165 (12%) were diagnosed as TBU [6]. Most of these diagnoses are based on expert ophthalmologist opinion and immunological evidence of TB exposure (a reactive tuberculin skin test [TST] or interferon gamma release assay), while others may have microbiological confirmation from non-ocular samples (eg, sputum culture) [7]. Microbiological confirmation of TB infection from ocular samples is uncommon, due to low specimen volumes affecting the sensitivity of TB culture and Polymerase Chain Reaction (PCR) [8]. While only those with *M. tuberculosis* directly isolated from ocular samples can be said to have definitively confirmed TBU, these patients are generally grouped with presumed TBU in clinical research due to the low incidence of TBU overall [9, 10]. Figure 1 shows a proposed model of clinical certainty regarding the diagnosis of TBU, depicting the increasing certainty with increasing evidence of TBU. While only cases with a positive culture from the eye can unequivocally be said to be confirmed TBU, other cases can be diagnosed with TBU on a spectrum of certainty.

Ophthalmologists use the location and phenotypic characteristics of uveitis to differentiate between likely aetiologies. The Standardisation of Uveitis Nomenclature working group has published standardised grading systems for many of the different anatomical features/phenotypes of uveitis, to assist with clinical research in this field [11]. The Collaborative Ocular Tuberculosis Study (COTS) group compiled a list of clinical features suggestive of TBU in 2017 [12]. This list was based on a literature review and opinion from expert ophthalmologist contributors to the study. The following clinical signs were thought to be suggestive of TB as the aetiology for uveitis:

1. Anterior uveitis (granulomatous or nongranulomatous), iris nodules, and ciliary body granuloma.
2. Intermediate uveitis (granulomatous or nongranulomatous with exudates in the pars plana, with or without snowballs).
3. Posterior and panuveitis, choroidal tubercle, choroidal granuloma, subretinal abscess, and serpiginous-like choroiditis.
4. Retinitis, retinal vasculitis (RV), neuroretinitis, optic neuritis, endogenous endophthalmitis, panophthalmitis, and scleritis [12].

Of these, posterior and panuveitis are the more frequent presentations of TBU, where these presentations account for up to 70% of all TBU [13]. This contrasts with the relative rareness of these forms of uveitis, where posterior and panuveitis usually only account for < 30% of uveitis presentations overall [14, 15]. As posterior and pan-uveitis have higher rates of significant and irreversible vision loss than other anatomical forms of uveitis, prompt initiation of effective treatment is therefore paramount for the preservation of sight [15]. The incidence of TB in certain settings can influence the level of diagnostic suspicion of TB as a causative pathogen, and awareness of this possible diagnosis, changing the likelihood of an individual receiving a diagnosis of presumed TB uveitis.

Two distinct types of posterior uveitis have also been described as being particularly suggestive of TB infection – serpiginous like choroiditis (SLC) [16] and Eales/ “Eales-like” retinal vasculitis, due to these presentations often occurring in patients with positive TB cultures from extraocular sites, or improvement after anti-TB therapy [12]. This results in a treatment dilemma for the clinician when patients present with one of these forms of uveitis with evidence of TB exposure, but no available samples (from the eye or elsewhere) to confirm TB infection, as the treatment of non-infective posterior/panuveitis is long term aggressive immunosuppression [17]. Given the risk of immunosuppression in patients with possible TB, there is a need for a better understanding of the risks and benefits of ATT (both for TB disease and TB infection) in these patients.

A previously published systematic review and meta-analysis of the role of ATT in the management of patients with presumed TBU examined articles published between 1990 and 2015 [18]. All but one of the studies included in this review were retrospective, and six of the included 28 studies used response to ATT as an inclusion criterion. Despite the heterogeneity of diagnostic criteria, ATT regimens used and duration of therapy, a meta-analysis was performed. The meta-analysis did not find a significant benefit nor a significant detrimental effect of ATT for presumed TBU. A subsequent large and multicentre retrospective case series reported by the same group found that ATT did not significantly alter treatment outcomes. However, the authors still recommend commencing ATT in patients with TBU [19], based on expert opinion.

Given the existing consensus for initiating ATT for TBU [2] in the absence of strong evidence to support this practise, a new and more extensive systematic review was performed to establish the current state of evidence regarding ATT in TBU, and to provide guidance and direction for future research.

## Methods

Analysis methods and inclusion criteria for this systematic review were specified in advance and documented in a protocol in compliance with the ‘Preferred Reporting Items for Systematic Reviews and Meta-Analyses’ (PRISMA) Statement [20]. The protocol for the systematic review was published on PROSPERO in September 2021 (PROSPERO 2021 CRD42021273379) [21]. The PRISMA checklist for this study is available as Appendix A.

### Search strategy

A systematic review of Medline, Embase, and Central databases without date restrictions was conducted on 20 June 2023 to identify primary research that evaluated the effect of antituberculosis therapy in people with uveitis and evidence of TB exposure. Medline and Embase were searched using the Ovid interface with the following search strategy:

1. Tuberculosis, Ocular/
2. exp Tuberculosis/ and exp Uveitis/
3. ((uveitis or panuveitis or (serpiginous adj3 choroidit*)) and (tubercul* or TB)).mp. [mp = title, abstract, original title, name of substance word, subject heading word, floating sub-heading word, keyword heading word, organism supplementary concept word, protocol supplementary concept word, rare disease supplementary concept word, unique identifier, synonyms]
4. 1 or 2 or 3

Central was searched with the strategy ("uveitis"):ti,ab,kw AND (tuberculosis):ti,ab,kw", with word variations included.

No filters or limits were used.

### Inclusion criteria

Studies were included if they met the following criteria:

1. Participants had clinical diagnosis of uveitis and evidence of TB exposure (positive TB IGRA or TST), AND
2. The study was published since 1966, AND
3. Reported outcomes of participants’ therapy, AND
4. Reported whether participants received ATT, AND
5. Detailed the nature of antitubercular therapy used.

Studies published in any language were included.

### Exclusion criteria

Studies were excluded if:

1. They were case reports, OR
2. Included fewer than 10 participants, OR
3. Participants had a positive microbiological diagnosis of TB (eg Ziehl–Neelsen smear, culture or PCR), OR
4. Reported on less than 6 months of follow up after initiation of therapies.
5. Studies where all participants had microbiological confirmation of TB disease from ocular or non-ocular specimens were excluded. Studies with a mix of positive and negative/not conducted microbiological results were excluded.

Studies were included where some participants had less than 6 months of follow up, however those individuals were not included in the analysis. Studies with fewer than 10 participants or where all participants had less than 6 months’ follow up were excluded.

Studies that were unable to be sourced were also excluded. Two reviewers, and librarians from two academic libraries (The Royal Melbourne Hospital Library and The University of Melbourne Library) all performed searches for studies before it was decided that they were unable to be sourced.

### Screening strategy

All studies that met the search criteria were transferred to Covidence systematic review software (Veritas Health Innovation, Australia, 2022).

Following automated removal of duplicate publications by Covidence, studies were screened for inclusion by two independent reviewers. Discrepancies between the two reviewers were resolved by consensus or by consultation with a third reviewer if consensus could not be reached.

Title and abstract screening was performed first, and studies that met the inclusion criteria from this stage proceeded to full text screening.

### Analysis

The following variables were extracted where reported: year of study commencement, country in which study was conducted, study design, duration of follow up, age range, tests confirming TB exposure (TST or IGRA), proportion of patients receiving topical, periocular and/or systemic immunosuppression, outcome at study completion, visual acuity at study completion, number of recurrences after treatment. One reviewer (JT) extracted this data, and no automation tools were used.

As a variety of outcome measures and follow up periods were anticipated, a random-effects meta-analysis was planned if sufficient homogeneity in available data was identified. If a meta-analysis was not possible, a narrative analysis would be performed.

The Newcastle–Ottawa scale was used to assess the quality of studies. This scale was chosen due to its design allowing for retrospective and non-randomised study designs [22]. One reviewer (JT) assessed risk of bias.

## Results

### Results of the literature search

**Fig. 2:**
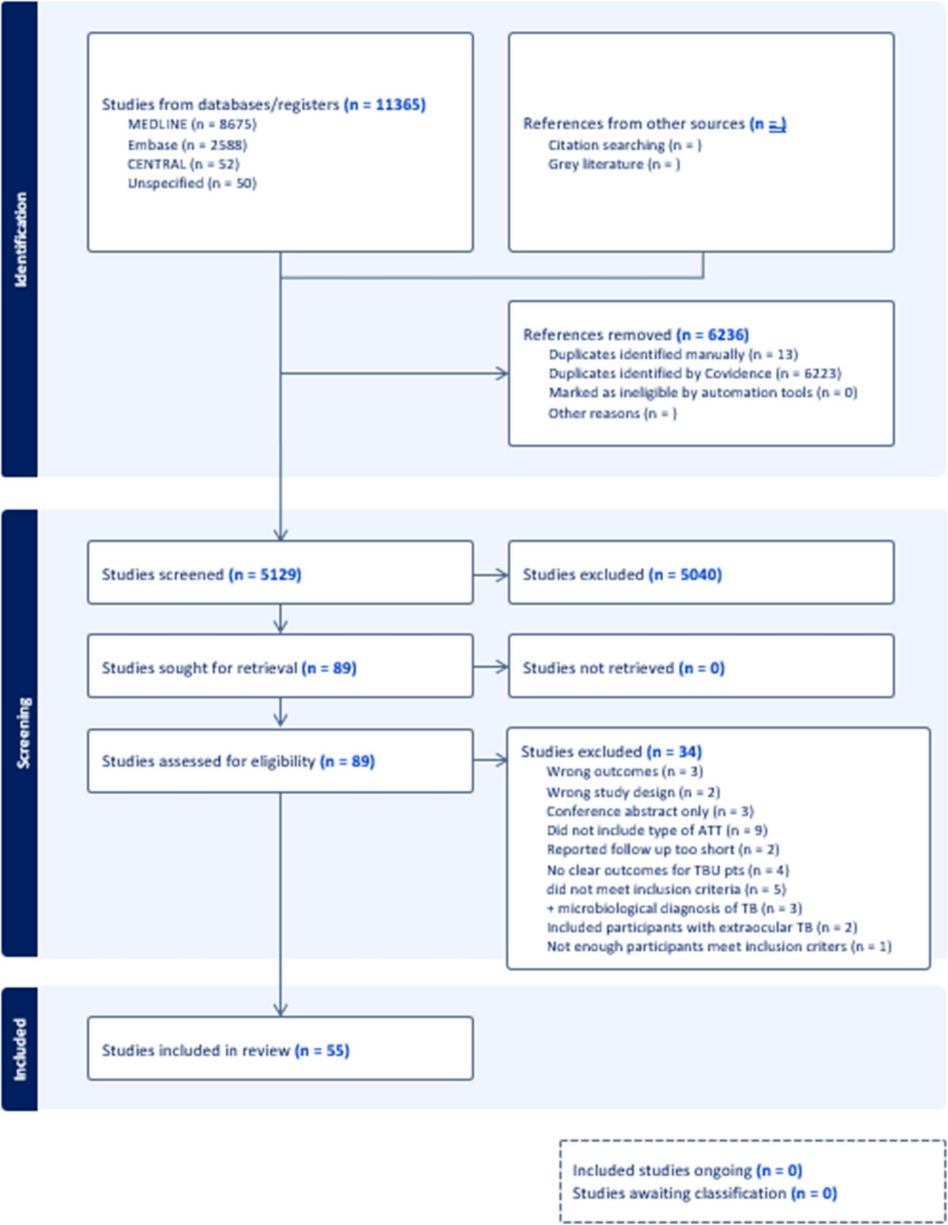
Flow chart of included studies

We identified 11365 studies, of which 6236 were excluded as duplicate publications. Of the remaining 5129 studies, 5040 studies were deemed irrelevant by title and abstract review. Of the remaining 90 studies, one was unable to be translated and therefore excluded, 34 were excluded as not meeting inclusion criteria (see Fig. 2 for details), leaving 55 studies included in the final analysis. Table 1 provides a complete review of the studies included and is available in the supplementary material.

### Definitions used

Significant variation in definitions used was found. Variations existed in diagnostic criteria, inclusion and exclusion criteria and outcome definitions.

Studies varied greatly in the type of TB therapy used, with multiple studies not differentiating between latent or active TB therapy. There was also variation in the definition of evidence of TB exposure, with some studies requiring chest X-Ray evidence of old TB or known contact with a TB case in addition to a positive TST or TB-IGRA. Multiple studies also included participants with a positive microbiological specimen from a non-ocular site. Exclusion of these individuals was sometimes difficult as they were not clearly identifiable from other participants, however the ophthalmological findings and predicted course is not anticipated to differ, and so should not have affected the outcomes in question. Studies varied in whether they used number of participants or number of eyes as their denominator. While most studies did not use standardised reporting for uveitis, 26 studies [9, 10, 19, 23–45] utilised the Standardised Uveitis Nomenclature (SUN) [11] and five studies [19, 30, 44, 46, 47] utilised the Standardised Nomenclature for Ocular Tuberculosis [48]. Outcomes reported varied widely, with 12 studies reporting visual acuity, 5 reporting clinical improvement or treatment response, 4 reporting treatment failure, 9 reporting number of recurrences, and 12 reporting mixed or multiple outcomes. The remaining 13 studies reported a variety of other outcomes, or other composite outcomes.

This resulted in large heterogeneity of studies extracted, which precluded meta-analysis. This informed the decision to proceed with narrative analysis rather than attempting meta-analysis.

### Study characteristics

There was heterogeneity in study designs included in the analysis. There were 7 prospective case series’, 3 prospective cohort series’, 1 retrospective case control study, 37 retrospective case series’, 6 retrospective cohort and 1 retrospective cross-sectional study identified. The dominance of retrospective and observational methodology is likely due to the uncommon nature of the diagnosis. Study size ranged from 11 to 447 participants. Recruitment periods varied from 4 months to 17 years, with retrospective methodologies permitting longer recruitment periods.

As expected, the location of studies was heavily weighted to TB endemic settings, with 2 studies being conducted in Australia/New Zealand, 3 in North America, 23 in Europe/the UK, 2 in Africa, 2 in the middle east and 21 in Asia. One study was conducted in South America, and one was multinational. Almost half the included participants came from studies conducted in Asia (1678/3838 participants), and only 12 of the studies were conducted in countries with an incidence of TB > 40/100 000 (as per 2023 WHO incidence data).

**Fig. 3:**
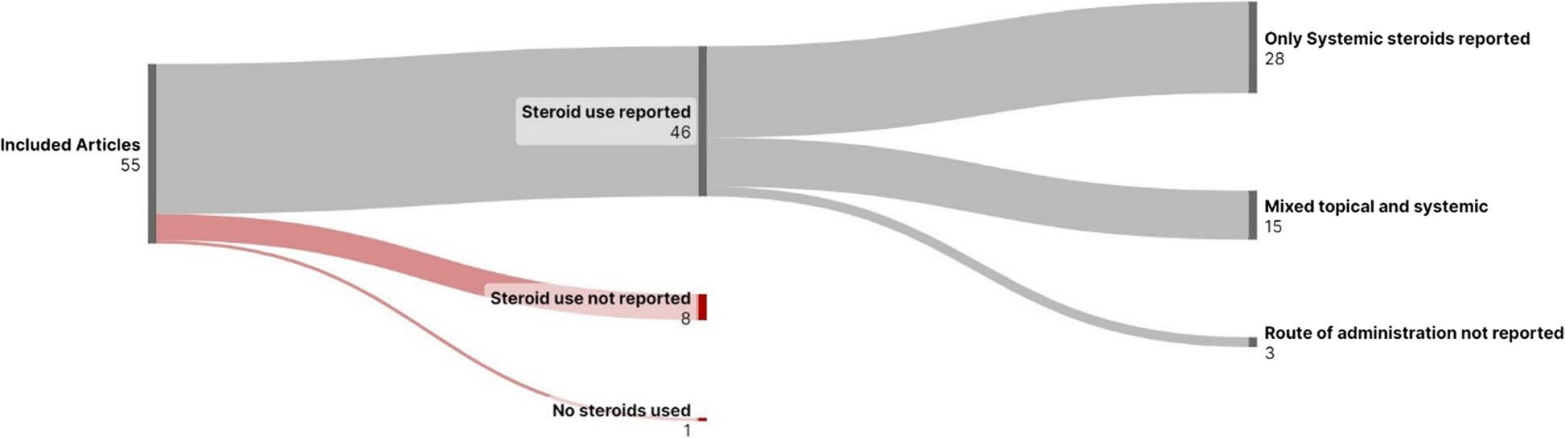
Reported steroid use

In studies that compared outcomes between those who received steroids alone and those who received steroid therapy plus ATT, there was a significant difference in the number of participants who received steroids in each group [49, 50]. This makes an analysis of the impact of steroid therapy very difficult. In most studies, participants who received ATT were less likely to receive steroid therapy. Figure 3 depicts the description of steroid use provided by the included studies.

**Fig. 4:**
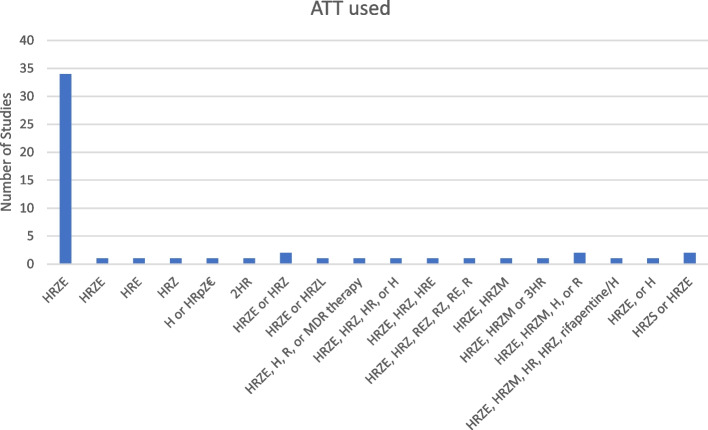
TB therapies used

Of the 3838 included participants, 687 (18%) did not receive ATT. Within those who did receive ATT, the regimens were highly varied both in terms of drugs used and in terms of durations of therapy. The most common regimen used was standard therapy of Isoniazid (H), Rifampicin (R), Ethambutol (E) and Pyrazinamide (Z). There was some substitution of fluoroquinolones for pyrazinamide in later studies, and two studies included participants who received rifapentine instead of rifampicin. There were no explicit descriptions of the use of second line drugs, likely in part due to the lack of microbiological confirmation precluding drug susceptibility testing in these patients. Many studies also do not differentiate between latent TB therapy and active TB therapy when grouping participants into those who received and those who did not receive TB therapy. This further confounds potential meta-analysis of the studies. Figure 4 shows the frequency of different ATTs described in the studies.

It should be noted that three studies appear to have used the same cohort of participants for their analyses [49, 51, 52]. All three studies describe exactly 43 participants who received moxifloxacin as part of their ATT regimen, with the same outcomes being reported in two out of the three studies, and with the same list of authors for all three. While the two studies authored by Agrawal et al. [49, 53] look at the same outcomes and make similar assertions in their conclusions, the study by Potter et al. [52] was designed to compare outcomes between those who received a fluoroquinolone in their regimen and those who did not.

### Study outcomes

Studies reporting visual acuity (VA) found that most participants improved or were stable over time. Very few studies reported outcomes for participants that did not receive ATT, however one study of participants who were managed with immunosuppressive therapy alone [42] found similar results, with 14/17 participants either having improvement in VA or stable VA.

Studies that reported treatment success, failure or recurrences did not show a clear difference between those who received ATT and those who did not.

### Risk of bias

Overall, methodological quality of studies was low. This demonstrates both the difficulty of conducting research in this field, and the difficulty clinicians face when trying to make decisions regarding management of patients with TBU. With the lack of high-quality data, most management decisions are made on expert opinion alone.

One of the included studies has a control arm for likely confounding variables. Oftentimes control arms contained participants with different patterns of uveitis (thought less likely to be due to TB), or participants with no evidence of TB exposure. As most studies are retrospective, they exclude participants who were prescribed TB therapy but did not complete the course, either due to non-adherence or intolerance. No studies include a description of outcomes for those who did not complete their ATT. In one study, 30% of participants prescribed TB therapy did not complete their course [50]. This cohort may have been a more representative group to compare to those who did complete their course of ATT, but instead they were excluded from the analysis and participants who were never prescribed ATT were used as the comparison, with all the risks of bias that this introduces.

## Discussion

This study highlights the significant heterogeneity of studies in the field of TB uveitis, and the lack of quality evidence in this area. This is in part due to the difficulties in conducting studies of a rare condition and a lack of standardised outcomes in this area of research.

While the search terms included studies published after 1966, many earlier studies were very difficult to locate. The earliest study included in the analysis was started in 1992, with the majority of the 17 unsourced studies being from earlier, non-English language publications. This has led to a bias of publications available in English, and accessible in full. As earlier studies may have included participants from prior to the widespread availability of ATT, information regarding the natural history of TBU without ATT may have been available in these studies.

A recurring issue with many of the studies included in this analysis is that they used a positive response to initiating ATT as one of the diagnostic criteria for TBU. Six studies used this criterion for inclusion, [38, 54–58]. This creates significant bias in the outcomes of studies, with a positive response to treatment being an inclusion criterion in the assessment of that treatment. Many studies also only included participants with uveitis and immunological evidence of TB exposure who had been started on ATT [25, 34, 37, 38, 46, 51, 57–60]. The exclusion of participants meeting these criteria who were not managed with ATT results in a dearth of data regarding other managements, or the natural history of the disease.

Very few of the included studies reported data on adverse effects of ATT. The side effects of ATT are significant. When reported, adverse effects were present in approximately 1/3 of TBU patients prescribed ATT [61]. When trying to establish the risk to benefit ratio of a therapy, it is very important to understand the risks involved. While rates of adverse events and serious adverse events from ATT in general are well understood [62], it would be beneficial to have a greater understanding of these risks in the uveitis population. In addition to the risk of adverse medication effects, there are other burdens of ATT. These burdens include the duration of therapy, the pill burden, the cost of therapy and potential lost wages, impacts on reporoductive choices, stigma and many other burdens. These burdens need to be included in the risk:benefit analysis of ATT for pateints with presumed TBU.

Our review did not undertake meta-analysis given the heterogeneity in interventions, settings and outcomes identified. We note that a meta-analysis with conceptual focus overlapping this review was recently published [63]. This review includes many of the same studies as this review, however, the authors provide a meta-analysis of the data. Despite confounding variables present in these studies, the authors conclude that the evidence supports ATT administration as improving visual acuity and reducing recurrences of inflammation in patients with TBU. Of the nine studies included in their analysis of inflammatory recurrence rates with and without TBU, three reported significant differences in baseline characteristics and steroid use between participants who received and did not receive ATT. Two of the included studies reported that participants receiving ATT were more likely to have evidence of TB exposure and were more likely to receive steroid therapy [50, 64]. A further study comparing outcomes between participants who received and did not receive ATT reported significant differences in the phenotype of uveitis between these groups [65]. One compared participants’ pre and post ATT outcomes, with all participants receiving ATT and significant time bias was introduced [28]. All were retrospective, with no controls regarding the decision to start ATT [28, 50, 64–70]. This review is limited by the inability to perform a meta-analysis, and the quality of the included studies.

None of the included studies were randomised. This lack of prospective, randomised data greatly impairs our understanding of the utility of ATT for TBU. Some studies with equivocal or weak evidence for the benefit of ATT for TBU make strong recommendations for ATT in this setting [19, 27]. As a result of this lack of prospective, randomised data, current recommendations do not have high levels of evidence to support them. When any therapy is strongly recommended, it becomes difficult to demonstrate the clinical equipoise required to ethically conduct randomisation for clinical trials. There is clearly a significant need for future randomised studies to improve our understanding of the utility of ATT in this setting. However, the rarity of TBU, and the limited resources in places with higher incidence makes this logistically challenging. Whilst the COTS initiative makes some attempt to standardise terminology in TBU, further steps are needed to strengthen clinical data such as a consensus regarding key outcome measures, follow up periods and reporting standards for the use of ATT and immunosuppressive therapy. Greater standardisation in such data collection and reporting would also assist in feasibility assessment for interventional clinical trials.

## Conclusion

This systematic review demonstrates the current evidence base regarding ATT for TBU. While most patients had a positive outcome associated with ATT in this setting, the quality of the data available does not allow for strong conclusions to be made with regards to the impact of ATT on disease progression, response or relapse risk. This systematic review illuminates the pressing need for further research into this sight-threatening condition, particularly prospective, randomised, or well controlled cohort studies. Despite the significantly limited feasibility of such studies, they may finally provide data that can inform high quality evidence-based guidelines.

## Supplementary Information

Supplementary Material 1.

Supplementary Material 2.

Supplementary Material 3.

## Data Availability

All data generated or analysed during this study are included in this published article and its supplementary material.
