# Supplementary material for: A systemic review of the utility of antituberculosis therapy for presumed tuberculous uveitis
Source: BMC Infect Dis. 2025 Jan 24;25:112. doi: 10.1186/s12879-024-10288-1 (PMC11761210; doi:10.1186/s12879-024-10288-1)
Supplement: Supplementary file 2 — Supplementary Material 2. [file 12879_2024_10288_MOESM2_ESM.docx]

Table 1: Characteristics of Included Studies

| Authors | Design | Year of commencement | Country | | Participants | | Duration of follow up | | TST/IGRA | | ATT | | Steroids | | Outcome measured | Outcome result BCVA | Outcome Result recurrences | Outcome Result Success |
| --- | --- | --- | --- | --- | --- | --- | --- | --- | --- | --- | --- | --- | --- | --- | --- | --- | --- | --- |
| Agarwal 2020^54^ | Retro.  case series | 2002-2019 | India | | 183 | | 6 mo. | | TST or IGRA | | 183 HRZE | | 183 sys | | Good or Poor outcome |  |  | 104 eyes good, 99 eyes poor |
| Agrawal 2015^51^ | Retro.  cohort | NR | UK | | 175 | | ≥ 6 mo. | | IGRA | | 122 HRZE, 43 HRZM, 10 H or R | | 120 sys, 116 top | | Treatment failure |  |  | 135 success, 32 failure |
| Agrawal 2016^49^ | Retr. cross sectional | NR | UK | | 213 | | 6 mo. | | IGRA | | 10 HR, 93 HRZE, 43 HRZM, | | 93 sys 157 top | | Treatment failure |  |  | ATT: 21.32%, no ATT: 29.87% |
| Agrawal 2017^55^ | Retro.  case series | NR | UK | | 77 | | ≥ 6 mo. | | IGRA | | 50 HRZE or HRZM or H or R | | 75% sys | | Effect size of ATT |  |  | Not significant in any subset |
| Agrawal 2020^19^ | Retro.  case series | 2004-2014 | Multinational | | 447 | | ≥ 1 year | | not described | | 382 HRZE | | 382 Sys | | Treatment failure |  |  | No ATT: 18%, ATT: 17% |
| Al-Mezaine 2008^56^ | Retro. case series | 1998-2006 | Saudi Arabia | | 51 | | mean 18.9 mo. | | TST | | 51 HRZE | | 51 sys | | VA | 53.4% eyes improved, 6.8% eyes worse, 39.8% eyes same |  |  |
| Al-Qarni 2019^41^ | Retro. Case series | 1996-2013 | Saudi Arabia | | 90 | | Mean 6.7 mo. | | TST | | 90 HRZE | | 90 sys | | VA | 67 eyes same, 14 eyes worse, 60 eyes better |  |  |
| Amara 2021^24^ | Pro. case series | 2016 | France | | 18 | | 12 mo. | | IGRA | | 2 HR(2 months) | | NR | | Cure and recurrence |  | No recurrences | 100% cure |
| Ang 2012^57^ | Retro. Case contol | 2000-2008 | | Singapore | | 182 | | ≥6 mo. | | TST | | 46 HRZE, 18 incomplete HRZE | | 141 sys | Recurrence |  | no ATT: 17/118 recurrence free, ATT 11/46 recurrence free |  |
| Ang 2016^36^ | Retro. case series | 2007-2012 | Singapore | | 53 | | ≥12 mo. | |  | | 36 HRZE | | 28 sys | | Treatment failure |  |  | ATT: 11/36, no ATT: 3/17 |
| Anibarro 2018^37^ | Retro. case series | 2006-2016 | Spain | | 24 | | median 41 mo. | | TST or IGRA | | 24 HRZE or HRZ | | 41.7% sys | | Treatment response |  |  | 22 eyes complete, 7 eyes partial r, 10 eyes failure or recurrence |
| Babu 2009^28^ | Retro. case series | 1997-2008 | India | | 51 | | ≥ 1 year | | TST | | 51 HRZE | | 46 but ROA NR | | Recurrence |  | 80.4% no recurrences |  |
| Bajema 2017^26^ | Retro. case series | 2010-2017 | USA | | 20 | | 6-12 mo. | | TST or IGRA | | 14 HRZE or HRZM, 1HR, 1 HRZ, 1 Rp/H | | 12 sys | | Improvement |  |  | ATT: 14/17 improved, no ATT: 1/3 improved |
| Bansal 2008^50^ | Retro. case series | 1991-2005 | India | | 360 | | ≥1 year | | TST | | 216 HRZE | | 360 sys or local | | Recurrence |  |  | ATT: 15.74% recurrences, no ATT: 46.53% recurrences |
| Bansal 2012^58^ | Retro. cohort | 2002-2010 | India | | 105 | | ≥9 mo. | | IGRA | | 93 HRZE | | 105 sysl | | Progression of ocular lesions, recurrence |  | ATT: 9/93 recurrences, no ATT: 9/12 recurrences | ATT: 12.9% progression, no ATT: 0% progression |
| Basu 2013^31^ | Retro. case series | 2008-2010 | India | | 106 | | ≥1 year | | TST | | 106 HRZE | | 106 ROA NR | | Progression of ocular inflammation |  |  | 24.5% progression |
| Bigdon 2022^42^ | Retro. Case series | 2016-2020 | Germany | | 17 | | Mean 28 mo. | | IGRA | | 0 | | 16 sys | | VA and remission | 6 same, 8 better, 3 worse |  | 14 complete remission, 3 incomplete remission |
| Brunner 2018^33^ | Retro. case series | 2012-2014 | Switzerland | | 12 | | mean 27.5 mo. | | IGRA | | 1 HRZE, 6 H | | 8 sys | | Reduction in ocular inflammation |  |  | ATT: 1/1 improvement, GCS/LTBT: 5/11 improved, 6/11 stable, 0/11 worse |
| Chung 2018^34^ | Retro. case series | 2000-2015 | Hong Kong | | 14 | | ≥1 year | | IGRA | | 14 HRZS or HRZE | | 8 sys | | Remission and VA | 6 same, 3 better, 5 worse | 13 remission |  |
| Cimino 2009^59^ | Retro. case series | NR | Italy and Switzerland | | 37 | | mean 30.4 mo. | | TST | | 37 HRE | | 37 sys, 12 perioc | | Recurrence and VA | 32/37 improvement with ATT | 3 recurrences post initiation of ATT |  |
| Conant 2016^60^ | Retro. case series | 2005-2013 | USA | | 39 | | average 16.3 mo. | | TST or IGRA | | 58% HRZE, 95% ATT (not specified) | | 9 sys | | VA and inflammation | 42/57 eyes stable or improved |  | 40/57 eyes inflammation improved |
| Connors 2019^61^ | Retro. case series | 2004-2014 | Canada | | 11 | | 4 years | | TST or IGRA | | 71% HR | | NR | | Clinical improvement (as per patient phone questionnaire) |  |  | 5 improved/resolved, 6 no change or worse |
| Damato 2017^27^ | Retro. case series | 2010-2014 | UK | | 41 | | ≥12 mo. | | TST or IGRA | | 27 HRZE, 9 HRZ, 3 REZ | | 37 sys | | Recurrence |  |  | 94% flare free at 6 months, 86% flare free at 12 months |
| Ducommun 2012^62^ | Retro. cohort | 1998-2004 | Switzerland | | 12 | | mean 4.5 years | | TST | | 5 H, 4HRpZ(E) | | 10 sys | | VA and relapses | no ATT: 1 better, 1 same, LTBT: 2 better, 2 same, 2 worse, ATBT: 2 same, 2 better | 2 relapses, one had no ATT, one had LTBT |  |
| Elangovan 2019^9^ | Retro. case series | 2014-2016 | Turkey | | 29 | | ≥ 6 mo. | | TST | | 28 HRZE, 1 2nd line therapy unspecified | | NR | | Favourable clinical response and BCVA | 6 worsened, 23 maintained or improved |  | 22 favourable clinical response |
| Fernandez-Zamora 2022 | Pros. Cohort | 2016-2019 | Brazil | | 72 | | Mean 29 mo. | | TST or IGRA | | 72 HRZE | | 33 sys, 17 top | | VA and cure | Mean BCVA improved from 0.57 to 0.4 |  | 58 cure or remission |
| Ghauri 2019^35^ | Pro. series | 2017 | Pakistan | | 40 | | 1 year | | TST (only 65% positive) | | 40 HRZE | | 3 sys | | Resolution |  |  | 32 resolved, 6 improved, 2 no change |
| Gupta 2011^63^ | Retro. series | 1992-2009 | India | | 84 | | ≥ 18 mo. | | TST | | 65 HRZE | | 19 sys | | Progression of uveitis |  | ATT: 11/65 progressed no ATT: 1/19 progressed |  |
| Jiang 2021^46^ | Retro. case series | 2008-2018 | China | | 66 | | ≥ 6 mo. | | TST or IGRA | | 66 HRZE | | 72.7% sys | | BCVA | 86.3% improved, 12.6% same, 1.1% worse |  |  |
| Kawali 2020^64^ | Retro. case series | 2010-2017 | India | | 18 | | ≥11 mo. | | TST or IGRA | | 15 HRZE | | with ATT: 15/15 sys, without ATT: 1/3 sys | | Recurrence and BVCA | no ATT: 1/3 improved, ATT: 10/15 improved | no ATT: 1/3, ATT 3/15 |  |
| Khochtali 2015^65^ | Retro. case series | 2009-2011 | Tunisia | | 38 | | 14 mo. | | TST or IGRA | | 33 HRZE | | 20 sys, 4 perioc | | Recurrence |  |  | 4 recurrences, ATT NR for these pts |
| Koubaa 2018^25^ | Retro. case series | 2006-2015 | Tunisia | | 12 | | mean 6.5 mo. | | TST | | 12 HRZE | | 11 sys | | Favourable outcome |  |  | 100% favourable |
| La Distia Nora 2014^39^ | Retro. cohort | NR | Netherlands | | 70 | | 1 year | | IGRA | | 32 HRZ | | NR | | VA | ATT: mean 46 line improvement, no ATT: mean 14 line improvement |  |  |
| LaCava 2020^66^ | Retro. case series | 2008-2018 | Italy | | 28 | | mean 3.2 years | | TST | | 18 HRZE, 7 H, 1 R, 2 MDR therapy | | 12 top, 8 sys, 2 perioc | | VA, recurrence, ocular complications | BCVA improved 0.7-0.8 with ATT, | 6 recurrences |  |
| Lal 1999^67^ | Pro. case series | 1992-1995 | India | | 14 | | 3 years | | TST | | 14 HRZE | | 0 | | Improvement |  |  | 11 improved, 1 mild response, 2 no improvement |
| Llorenç 2012^29^ | Pro. case series | 2009-2011 | Spain | | 103 | | ≥ 6 mo. | | TST and IGRA | | 37 HRZE | | 103 sys | | Recurrence |  | ATT: 33/37 recurrence free |  |
| Llorenc 2020^10^ | Retro. cohort | 2005-2017 | Spain | | 82 | | median 36 mo. | | TST or IGRA | | 51 HRZE | | 46 sys | | loss of >=2 lines of VA | OR 0.13, 95% CI 0.04 to 0.37; p < 0.001 |  |  |
| Manousaridis 2013^68^ | Retro. case series | 2002-2011 | UK | | 18 | | ≥ 6 mo. | | TST or IGRA | | 17 HRZE | | 3 sys, 6 perioc | | VA | ATT: 4/17 same, 11/17 better, 2/17 worse,  no ATT: 1/1 better |  |  |
| Mao 2014^69^ | Pro. case series | 2011-2013 | China | | 38 | | 1 year | | TST or IGRA | | 38 HRZE | | NR | | Recurrence |  |  | No recurrences |
| Mora 2015^43^ | Retro. Case series | 2000-2013 | Italy and France | | 30 | | Mean 23 mo. | | TST or IGRA | | 13 HRZE, 9 3-drug, 7 2-drug, 1H | | NR | | Recurrance |  | ATT not associated with fewer recurrences |  |
| Nahon-Esteve 2020^30^ | Retro. case series | 2004-2018 | France | | 18 | | 9 mo. | | TST or IGRA | | 18 HRZE or HRZ | | 5 sys, 18 top, 2 intraoc, 4 perioc | | Presence of inflammation |  |  | 4/18 active inflammation |
| Ng 2017^70^ | Retro. case series | 2007-2014 | NZ | | 39 | | 36 mo. | | TST or IGRA | | 30 HRZE (27 completed) | | NR | | Remission |  |  | ATT: 16/24, no/incomplete ATT: 4/11 |
| Oray 2017^71^ | Retro. case series | 1995-2013 | Turkey | | 28 | | 1 year | | TST or IGRA | | 17 HRZE | | 28 ROA NR | | BCVA | no significant difference |  |  |
| Potter 2016^52^ | Retro. cohort | 2009-2013 | UK | | 60 | | 6 mo. | | IGRA | | 43 HRZM, 17 HRZE | | 37 sys, 6 top, 5 perioc, 17 sys+top, | | VA | 32 same, 18 improved, 6 worse |  |  |
| Sanghvi 2011^7^ | Retro. case series | 1992-2007 | UK | | 23 | | 1 year | | TST | | 23 HRZE | | 16 sys | | VA | 5 same, 15 improved, 4 worse |  |  |
| Shahidatul-Adha 2017^72^ | Retro. case series | 2011-2016 | Malaysia | | 34 | | 1 year | | TST | | 34 HRZE or HRZS | | 100% top 67.6% sys | | BCVA | reduction in number of participants with BCVA 6/60 from 22 to 18 |  |  |
| Shirley 2020^73^ | Pro. series | 2016-2017 | UK | | 48 | | 1 year | | IGRA (+14 TST) | | 71% HRZE, 29% not specified | | 60% sys | | Questionnaire (ophthalmologist)/BCVA | 0.1 LogMAR improvement |  | 95.8% improvement |
| Teixeira-Lopes 2018^74^ | Retro. case series | 2012-2015 | Portugal | | 39 | | 29.9 mo. | | TST or IGRA | | 39 HRZE or HRZL | | NR | | Improvement (as per ophthalmologist) |  |  | 91.40% |
| Tognon 2014^40^ | Pro. cohort | 2007-2010 | Italy | | 45 | | 2 years | | IGRA | | 45 HRZE | | 4 sys | | VA and recurrence | Average VA stable in 67/78 eyes | 8 recurrences out of 78 eyes |  |
| Tomkins-Netzer 2018^23^ | Retro. cohort | 2007-2015 | UK and Australia | | 129 | | up to 16 years | | TST or IGRA | | 89 HRZE, 40 no ATT | | 138 top, 30 perioc, 113 sys | | BCVA and recurrence | ATT: 5.3 ± 1.5 letters No ATT: 3 ± 3.1 letters, P = .16 | 29.5% ATT vs 48.2% no ATT, OR 0.5, 95% CI 0.31-0.83, P = .006 |  |
| Tsui 2021^44^ | Retro. Case series | 2014-2019 | Hong Kong | | 19 | | Min. 6 mo. | | TST or IGRA | | 18HRZE | | 12 sys, 9 top | | Failure |  |  | 6/18 worsened, 4/18 had treatment failure |
| Urzua 2017^38^ | Retro. case series | 2002-2012 | Chile and Spain | | 35 | | mean 64.1 mo. | | TST | | 4 HRZE, 29 HRZ, 2 HRE | | 10 sys | | VA | 36 eyes VA <20/50,  17 eyes VA <20/200 |  |  |
| Yasaratne 2010^75^ | Pro. case series | 2006-2008 | Sri Lanka | | 23 | | median 14 mo. | | TST | | 17 HRZE | | 18 sys | | VA | ATT: 53% of pts VA improved |  |  |

Notes: NR = Not Reported, ROA = Route of Administration, Retro. = Retrospective, Pro. – Prospective, H – Isoniazid, R = Rifampicin, Z = Pyrazinamide, E = Ethambutol, M = Moxifloxacin, Rp = Rifapentine, S = Streptomycin, L = Levofloxacin, MDR = Multi Drug Resistant, VA = Visual Acuity, BCVA = Best Corrected Visual Acuity, Sys = Systemic, Top = topical, Perioc = periocular, intraoc = intraocular, GCS = Glucocorticosteriods, LTBT = Latent TB therapy, ATBT = active TB therapy, pts = patients
