# Supplementary material for: A systemic review of the utility of antituberculosis therapy for presumed tuberculous uveitis
Source: BMC Infect Dis. 2025 Jan 24;25:112. doi: 10.1186/s12879-024-10288-1 (PMC11761210; doi:10.1186/s12879-024-10288-1)
Supplement: Supplementary file 3 — Supplementary Material 3. [file 12879_2024_10288_MOESM3_ESM.docx]

## Risk of Bias

Table 2: Newcastle-Ottawa Quality Assessment Results

|  | Selection | | | | Comparability | Outcome | | | Total | Rating |
| --- | --- | --- | --- | --- | --- | --- | --- | --- | --- | --- |
| Authors | Representativeness of the exposed cohort | Selection of the nonexposed cohort | Ascertainment of exposure | Demonstration that outcome of interest was not present at start of study | Comparability of cohorts on the basis of design or analysis | Assessment of outcome | Was follow up long enough for outcomes to occur | Adequacy of follow up of cohorts |  | |
| Oray 2017^71^ | 1 | 1 | 1 | 0 | 0 | 1 | 1 | 1 | 6 | Moderate risk of bias |
| Shahidatul-Adha 2017^72^ | 1 | 0 | 1 | 1 | 0 | 1 | 1 | 1 | 6 | Moderate risk of bias |
| Jiang 2021^46^ | 1 | 0 | 1 | 1 | 0 | 1 | 1 | 1 | 6 | Moderate risk of bias |
| Tomkins-Netzer 2018^23^ | 1 | 1 | 1 | 1 | 1 | 1 | 1 | 0 | 7 | Low risk of bias |
| Lal 1999^67^ | 1 | 0 | 0 | 1 | 0 | 0 | 1 | 0 | 3 | High risk of bias |
| Teixeira-Lopes 2018^74^ | 1 | 0 | 1 | 1 | 0 | 1 | 1 | 1 | 6 | Moderate risk of bias |
| Connors 2019^61^ | 1 | 1 | 1 | 1 | 0 | 0 | 1 | 0 | 5 | Moderate risk of bias |
| Amara 2021^24^ | 1 | 1 | 1 | 1 | 0 | 1 | 1 | 0 | 6 | Moderate risk of bias |
| Agrawal 2017^55^ | 1 | 1 | 1 | 1 | 0 | 1 | 1 | 0 | 6 | Moderate risk of bias |
| Elangovan 2019^9^ | 1 | 0 | 1 | 1 | 0 | 0 | 1 | 1 | 5 | Moderate risk of bias |
| Koubaa 2018^25^ | 1 | 0 | 1 | 1 | 0 | 1 | 1 | 1 | 6 | Moderate risk of bias |
| Agarwal 2020^54^ | 1 | 0 | 1 | 1 | 0 | 1 | 1 | 1 | 6 | Moderate risk of bias |
| Bajema 2017^26^ | 1 | 1 | 1 | 1 | 0 | 1 | 1 | 1 | 7 | Low risk of bias |
| Llorenc 2020^10^ | 1 | 1 | 1 | 1 | 0 | 1 | 1 | 1 | 7 | Low risk of bias |
| Damato 2017^27^ | 1 | 0 | 1 | 1 | 0 | 1 | 1 | 0 | 5 | Moderate risk of bias |
| Babu 2009^28^ | 1 | 0 | 1 | 1 | 0 | 1 | 1 | 1 | 6 | Moderate risk of bias |
| Llorenç 2012^29^ | 1 | 1 | 1 | 1 | 0 | 0 | 1 | 1 | 6 | Moderate risk of bias |
| Nahon-Esteve 2020^30^ | 1 | 0 | 1 | 1 | 0 | 1 | 1 | 0 | 5 | Moderate risk of bias |
| Basu 2013^31^ | 1 | 0 | 1 | 1 | 0 | 1 | 1 | 1 | 6 | Moderate risk of bias |
| Bansal 2012^58^ | 1 | 1 | 1 | 1 | 0 | 1 | 1 | 0 | 6 | Moderate risk of bias |
| Gupta 2011^63^ | 1 | 1 | 1 | 1 | 0 | 1 | 1 | 1 | 7 | Low risk of bias |
| Shirley 2020^73^ | 1 | 0 | 1 | 1 | 0 | 0 | 1 | 0 | 4 | Moderate risk of bias |
| Khochtali 2015^65^ | 1 | 0 | 1 | 0 | 0 | 1 | 1 | 1 | 5 | Moderate risk of bias |
| Mao 2014^69^ | 1 | 0 | 1 | 0 | 0 | 0 | 1 | 0 | 3 | High risk of bias |
| Kawali 2020^64^ | 1 | 0 | 1 | 1 | 0 | 1 | 1 | 1 | 6 | Moderate risk of bias |
| Hedayatfar 2011^32^ | 1 | 1 | 1 | 1 | 0 | 1 | 1 | 1 | 7 | Low risk of bias |
| Bansal 2008^50^ | 1 | 1 | 1 | 1 | 0 | 1 | 1 | 0 | 6 | Moderate risk of bias |
| Cimino 2009^59^ | 1 | 0 | 1 | 1 | 0 | 1 | 1 | 1 | 6 | Moderate risk of bias |
| Brunner 2018^33^ | 1 | 0 | 1 | 1 | 0 | 1 | 1 | 1 | 6 | Moderate risk of bias |
| Ng 2017^70^ | 1 | 1 | 1 | 1 | 0 | 1 | 1 | 1 | 7 | Low risk of bias |
| Chung 2018^34^ | 1 | 0 | 1 | 1 | 0 | 1 | 1 | 1 | 6 | Moderate risk of bias |
| Ghauri 2019^35^ | 1 | 0 | 1 | 1 | 0 | 1 | 1 | 1 | 6 | Moderate risk of bias |
| Ang 2018^36^ | 1 | 1 | 1 | 1 | 0 | 1 | 1 | 1 | 7 | Low risk of bias |
| Agrawal 2015^51^ | 1 | 0 | 1 | 1 | 0 | 1 | 1 | 1 | 6 | Moderate risk of bias |
| Agrawal 2016^49^ | 1 | 0 | 1 | 1 | 0 | 1 | 1 | 1 | 6 | Moderate risk of bias |
| Agrawal 2020^19^ | 1 | 1 | 1 | 1 | 0 | 1 | 1 | 1 | 7 | Low risk of bias |
| Anibarro 2018^37^ | 1 | 0 | 1 | 1 | 0 | 1 | 1 | 1 | 6 | Moderate risk of bias |
| Manousaridis 2013^68^ | 1 | 1 | 1 | 1 | 0 | 1 | 1 | 0 | 6 | Moderate risk of bias |
| Sanghvi 2011^7^ | 1 | 0 | 1 | 1 | 0 | 1 | 1 | 0 | 5 | Moderate risk of bias |
| Yasaratne 2010^75^ | 1 | 0 | 1 | 1 | 0 | 1 | 1 | 1 | 6 | Moderate risk of bias |
| Urzua 2017^38^ | 1 | 0 | 1 | 0 | 0 | 1 | 1 | 1 | 5 | Moderate risk of bias |
| Al-Mezaine 2008^56^ | 1 | 0 | 1 | 1 | 0 | 1 | 1 | 1 | 6 | Moderate risk of bias |
| Potter 2016^52^ | 1 | 0 | 1 | 1 | 0 | 1 | 1 | 1 | 6 | Moderate risk of bias |
| La Distia Nora 2014^39^ | 1 | 1 | 1 | 1 | 0 | 1 | 1 | 1 | 7 | Low risk of bias |
| Conant 2016^60^ | 1 | 0 | 1 | 1 | 0 | 1 | 1 | 1 | 6 | Moderate risk of bias |
| Tognon 2014^40^ | 1 | 0 | 1 | 1 | 0 | 1 | 1 | 1 | 6 | Moderate risk of bias |
| Ducommun 2012^62^ | 1 | 0 | 1 | 1 | 0 | 1 | 1 | 1 | 6 | Moderate risk of bias |
| LaCava 2020^66^ | 1 | 0 | 1 | 1 | 0 | 1 | 1 | 1 | 6 | Moderate risk of bias |
| Al-Qarni 2019^41^ | 1 | 0 | 1 | 0 | 0 | 1 | 1 | 1 | 5 | Moderate risk of bias |
| Bigdon 2022^42^ | 1 | 0 | 1 | 0 | 0 | 1 | 1 | 1 | 5 | Moderate risk of bias |
| Fernandez Zamora 2022^47^ | 1 | 0 | 1 | 0 | 0 | 1 | 1 | 1 | 5 | Moderate risk of bias |
| Ang 2016^45^ | 1 | 0 | 1 | 0 | 0 | 1 | 1 | 1 | 5 | Moderate risk of bias |
| Mora 2015^43^ | 1 | 0 | 1 | 0 | 0 | 1 | 1 | 1 | 5 | Moderate risk of bias |
| Multani 2020^76^ | 1 | 0 | 1 | 0 | 0 | 1 | 1 | 1 | 5 | Moderate risk of bias |
| Tsui 2021^44^ | 1 | 0 | 1 | 0 | 0 | 1 | 1 | 1 | 5 | Moderate risk of bias |
